# Reinforcement learning approach to control an inverted pendulum: A general framework for educational purposes

**DOI:** 10.1371/journal.pone.0280071

**Published:** 2023-02-13

**Authors:** Sardor Israilov, Li Fu, Jesús Sánchez-Rodríguez, Franco Fusco, Guillaume Allibert, Christophe Raufaste, Médéric Argentina

**Affiliations:** 1 Université Côte d’Azur, CNRS, INPHYNI, Valbonnes, France; 2 Université Côte d’Azur, CNRS, I3S, Sophia Antipolis, France; 3 Laboratory of Fluid Mechanics and Instabilities, École Polytechnique Fédérale de Lausanne, Lausanne, Switzerland; 4 Institut Universitaire de France (IUF), Paris, France; Nankai University, CHINA

## Abstract

Machine learning is often cited as a new paradigm in control theory, but is also often viewed as empirical and less intuitive for students than classical model-based methods. This is particularly the case for reinforcement learning, an approach that does not require any mathematical model to drive a system inside an unknown environment. This lack of intuition can be an obstacle to design experiments and implement this approach. Reversely there is a need to gain experience and intuition from experiments. In this article, we propose a general framework to reproduce successful experiments and simulations based on the inverted pendulum, a classic problem often used as a benchmark to evaluate control strategies. Two algorithms (basic Q-Learning and Deep Q-Networks (DQN)) are introduced, both in experiments and in simulation with a virtual environment, to give a comprehensive understanding of the approach and discuss its implementation on real systems. In experiments, we show that learning over a few hours is enough to control the pendulum with high accuracy. Simulations provide insights about the effect of each physical parameter and tests the feasibility and robustness of the approach.

## Introduction

Inverted pendulums—also known as “*cart-pole*” apparatuses—belong to simple type of system that have a long history in the field of mechanics and dynamical systems [1, 2]. Their dynamics is described by a set of mathematical equations that are simple to derive, while still featuring interesting properties such as nonlinearity and under-actuation. This makes an inverted pendulum a perfect candidate to benchmark and showcase new control algorithms before deploying them on more complex systems such as quadrotors or humanoid robots [3]. In addition, given the simplicity required to build an experimental prototype, cart-pole systems are very well-suited for teaching a wide variety of topics, ranging from Lagrangian mechanics to control theory. Indeed, the literature includes numerous examples of low-cost pendulums designed and built with the purpose of teaching one or more subjects to undergraduates [4–6].

In this article, we aim at controlling an inverted pendulum in its unstable position, by reinforcement learning (RL). This machine learning method has shown great interest in many applications such as playing games [7, 8] and system controlling [9–11], and focuses on how agents perform actions in an environment so as to maximize some notion of cumulative reward [12]. The advantage of RL is that it avoids modeling the dynamics involved, unlike in model-based approaches [13, 14].

Many numerical studies have implemented an inverted pendulum virtual environment as a benchmark to test RL algorithms [15–22], but to our knowledge, there is no study that provides successful RL implementations in experiments. First, except for a few studies that have discussed non ideal systems [16, 17], most of these numerical implementations discard the effects associated to realistic (and thus more complex) control methods: in experiments, the control of the cart is subject to delay, hysteresis, biases and noise that can significantly alter the learning process. Second, most of the existing virtual environments consider only motion of the pendulum in a small angle range around the upward and unstable position and do not treat the whole control from the downward and stable position as expected in experiments. This ambition makes the control task significantly more difficult.

The goal of the article is twofold. First, we expose the basic ingredients to build an intuition about RL approaches. We focus here on Q-learning and Deep Q-Network approaches to give insights about the implementation and the conditions of successful controls. Simulations with a virtual environment are provided to test the feasibility of the two approaches as well as to probe the effect of physical parameters that can not be easily tuned in experiments. Second, we provide all the material to perform experiments. This paper is accompanied by an open-source code repository which allows to replicate all the approaches presented here [23]. It includes detailed instructions to build the prototype used in this work, configure its software interface and implement several controllers.

## Modelling the inverted pendulum and the controller

**Fig 1:**
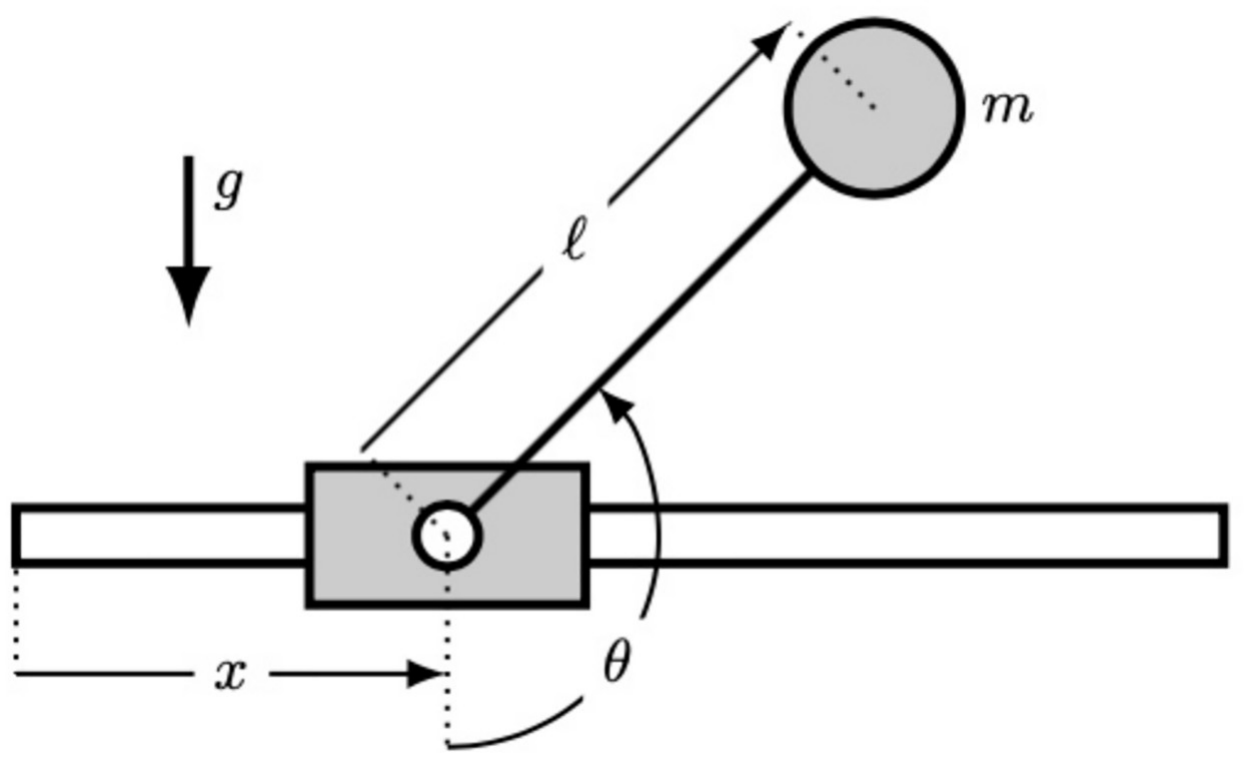
Sketch of the inverted pendulum.

We assume a mass *m* located at the end of a massless rigid rod of length ℓ and subjected to gravity *g*. Its other extremity is free to rotate on a motorized cart located at abscissa *x*(*t*). The angle *θ*(*t*) separating the rod to the downward vertical direction, as shown in Fig 1, follows the dynamics of a damped oscillator in the absence of cart motion. If the cart is actuated, the dynamics is driven by the equation:

$$\begin{array}{c} \ddot{\theta }+k_{v}\dot{\theta }+\omega^{2}\;\text{sin}\;\theta +\frac{\ddot{x}}{\ell }\;\text{cos}\;\theta =0, \end{array}$$ (1)

with $\omega =\sqrt{g/\ell }$ the natural frequency of the pendulum and *k*_*v*_ a viscous friction coefficient [1].

The purpose is to stabilize the pendulum in its unstable equilibrium position *θ* = *π* by controlling the motion of the cart only, which is itself driven by a target velocity provided by a controller. Unlike ideal systems implemented in virtual environments, experimental systems need to account for delay, hysteresis and biases between the target and measured values. For the present setup, the cart velocity $\dot{x}$ and the control velocity ${\dot{x}}_{c}$ are linked through the equation:

$$\begin{array}{c} \ddot{x}=\frac{1}{\tau }({\dot{x}}_{c}-\dot{x})-f_{c}\;\text{sign}\;\left(\dot{x}\right)-f_{d}. \end{array}$$ (2)

The first term on the right-hand side models the motorized cart with *τ*, a relaxation time scale to account for the linear dynamics. *f*_*c*_ and *f*_*d*_ are two coefficients to account for the asymmetric dry friction acting on the motorized base. In experiments, the cart target velocity ${\dot{x}}_{c}$ is proportional to the applied voltage *U*:

$$\begin{array}{c} {\dot{x}}_{c}=k_{U}U, \end{array}$$ (3)

where *k*_*U*_ is a constant. The cart is constrained to move on a track of length 2*x*_*max*_.

## Controlling the pendulum using reinforcement learning

RL exploits the framework of Markov Decision Process (MDP), which is an extension of the Markov process. There are four components in MDP: a set of states, a set of actions, a reward and a policy. We refer to the internal decision maker who uses a RL algorithm as an agent, and the whole physical system as the environment. During the learning process, the agent evolves in an environment and tries to maximize its cumulative reward. At each time step, the state of the agent is assessed and an action is performed. After the actuation, the environment provides a new state and a reward. The choice of the action follows the policy *π*(*a*|*s*) which is the probability of taking action *a* while in state *s*. The objective in RL is to determine the best policy π*(*a*|*s*) for the agent, that maximizes the cumulative reward.

For the cart-pole problem, at each time step *t*_*i*_ = *i*Δ*t*, the state *s*_*i*_ is given by the pendulum’s orientation *θ*(*t*_*i*_) and its angular velocity $\dot{\theta }\left(t_{i}\right)$, as well as the cart’s position *x*(*t*_*i*_) and velocity $\dot{x}\left(t_{i}\right)$, *i.e*.:

$$\begin{array}{c} s_{i}=\left(\theta \left(t_{i}\right),\dot{\theta }\left(t_{i}\right),x\left(t_{i}\right),\dot{x}\left(t_{i}\right)\right). \end{array}$$ (4)

**Fig 2:**
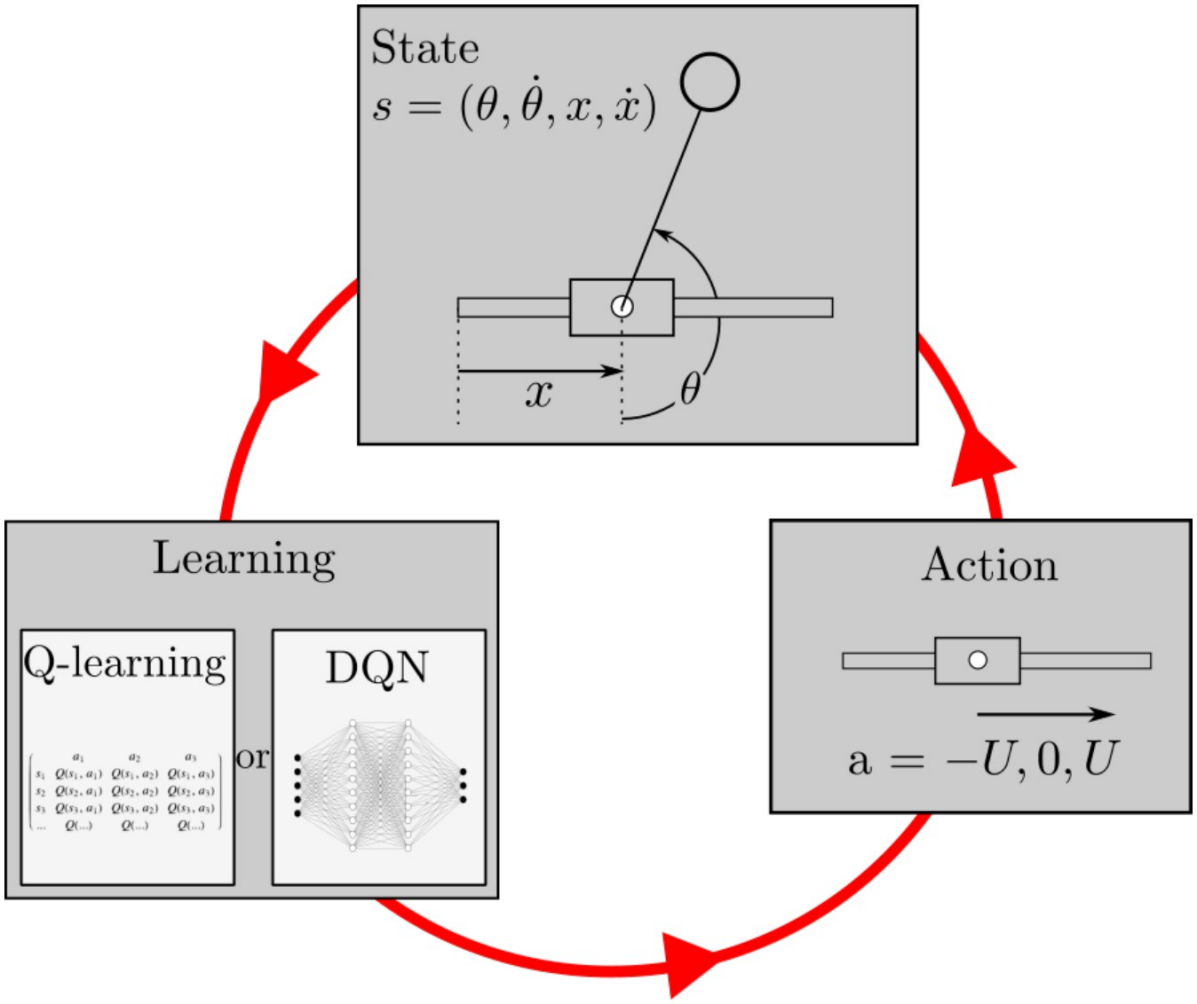
RL learning process. The state of environment *s* is measured and given to the agent. The agent updates it policy and accordingly choose the action *a* for the next step among −*U*, 0 or *U*. After sampling time, the state *s* evolves and the cycle continues. In our case, $s=(\theta ,\dot{\theta },x,\dot{x})$. The policy is updated by updating the action-value function for Q-Learning and DQN, using a so-called Q-table and artificial neural networks, respectively.

Here *i* is the time step number and Δ*t* is the time interval between two successive state observations (or between two successive actions). According to the policy *π*(*a*|*s*), the agent chooses and executes an action *a*_*i*_ which controls the cart movement for a given state *s*_*i*_. This action changes the agent’s state *s*_*i*_ to *s*_*i*+1_, and the environment provides a reward *r*_*i*+1_ related to the proximity of the pendulum to its unstable position. This process is then iterated at stage *i*+1: the loop is depicted in Fig 2. In order to construct the policy *π*(*a*|*s*), it is essential to estimate a return function *R*_*i*_, designed as the discounted cumulative reward:

$$\begin{array}{c} R_{i}=r_{i}+\gamma r_{i+1}+\gamma^{2}r_{i+2}+\gamma^{3}r_{i+3}+..., \end{array}$$ (5)

where 0 < *γ* < 1 is the discount factor which measures the importance of the future unitary reward in computing the expected cumulative reward. Since the cumulative reward depends on the states *s*_*i*_, *s*_*i*+1_, *s*_*i*+2_, … and the actions *a*_*i*_, *a*_*i*+1_, *a*_*n*+2_, …, one can define an action-value function *Q*(*s*_*i*_, *a*_*i*_) (*Q* refers to Quality) which computes the expected cumulative reward at the state *s*_*i*_ when performing the action *a*_*i*_:

$$\begin{array}{c} Q\left(s_{i},a_{i}\right)=E\left[R_{i}|\left(s_{i},a_{i}\right)\right]. \end{array}$$ (6)

This function could be the basis for constructing an optimal policy. For example a greedy policy will always select the best action a* = argmax_*a*_*Q*(*s*_*i*_, *a*) for an agent in state *s*_*i*_.

The learning process consists in visiting a large number of states and taking various actions, and to compute the reward expectation (6). However, it is usually time consuming and very difficult, if not impossible, to travel through all the states and actions to accurately determine the action-value function *Q*(*s*, *a*), as it is necessary to sample the state and the action spaces to accumulate statistics for the rewards. In addition, a control task could be infinitely long so it is not practical to wait to the end of the experiment and measure the cumulative reward, and update the function Q. In the MDP framework, one can rewrite Eq 6 as [12]:

$$\begin{array}{c} Q\left(s_{i},a_{i}\right)\approx r_{i}+\gamma Q\left(s_{i+1},a_{i+1}\right). \end{array}$$ (7)

Here we use the reward *r*_*i*_ after a sampled action *a*_*i*_ to represent the expected immediate reward, and *γQ*(*s*_*i*+1_, *a*_*i*+1_) to represent the cumulative discounted future reward. In order to determine the action-value function, the agent interacts constantly with the environment during the learning phase and update its *Q* function. This function can be updated through an iterative procedure:

$$\begin{array}{c} Q\left(s_{i},a_{i}\right)\leftarrow Q\left(s_{i},a_{i}\right)+\alpha \Delta Q, \end{array}$$ (8)

which is similar to the Euler scheme for numerically integrating differential equation $\dot{Q}=\Delta Q$, where *α* plays the role of a time step. This is the idea of what is called the temporal differencing (TD) approach [12]. By defining Δ*Q* = Q* − *Q*, we know that the differential equation will drive *Q* to the target Q*. The idea of the Q-Learning algorithm is to hypothesize that:

$$\begin{array}{c} Q^{*}\approx r_{i}+\gamma \;\underset{a^{\prime }}{\text{max}}\;Q\left(s_{i+1},a^{\prime }\right), \end{array}$$ (9)

with *a*′ being the accessible actions at state *s*_*i*+1_, which is consistent to the definition (7). It models that an approximation of the cumulative expected reward is the reward *r*_*i*_ plus the discounted cumulative reward at step *i* + 1 by taking the best action $a_{i+1}^{*}=\text{argmax}\left(Q\left(s_{i+1},a^{\prime }\right)\right)$. To summarize, the Q-learning iterative procedure writes [24, 25]:

$$\begin{array}{c} Q\left(s_{i},a_{i}\right)\leftarrow Q_{(}s_{i},a_{i})+\alpha (r_{i}+\gamma \;\underset{a^{\prime }}{\text{max}}\;Q\left(s_{i+1},a^{\prime }\right)-Q_{(}s_{i},a_{i})), \end{array}$$ (10)

where the parameter *α* measures the learning rate. The effect of the discount factor *γ* becomes even clearer: as it tends to zero, the learning agent only takes into account the immediate reward, while as *γ* is nonzero, the agent integrates future rewards in the learning phase. With this iterative approach, the agent learns while it evolves in the environment.

Q-learning employs an *ϵ*-greedy policy during the learning process. It chooses the best action most of the time, and slightly explore the consequences of randomly taken actions: at each time step, a random number *N*_*R*_ is drawn, if *N*_*R*_ < *ϵ* < 1, a random action will be chosen, otherwise the greedy policy will be applied.

To store the expectation of the cumulative reward, the Q-Learning algorithm uses a Q-table that covers the whole state space and action space. This object takes the form of a huge matrix of dimension *N*_*s*_ × *N*_*a*_, where *N*_*s*_ is the number of discretized states, and *N*_*a*_ is the number of possible actions (*N*_*a*_ = 3 here). This representation already underlines the limitation of this approach, because of the finite size of memory of modern computers.

To overcome this obstacle, it appears necessary to exploit a more efficient function approximator. Deep Q-Network (DQN) [7] is a reinforcement learning algorithm based on the Q-learning approach that takes advantage of neural networks in place of the matrix “Q-table” to approximate the true “action-value” function. Neural networks provide an effective way to approximate *Q*(*s*, *a*), because they can incorporate non-linearity and aggregate among the states due to the interconnection between the neighboring layers of the neural net. This leads to a more efficient action-value approximations. In addition, to stabilize the learning process and obtain more reliable results, DQN also employs a number of additional techniques that we summarize in S1 File.

In the next section, we perform the control task on a real system and we demonstrate the capacity of both Q-learning and DQN algorithms for the full control including swing-up and stabilization of the inverted pendulum. We first present in details our RL environment; then we discuss the limitations of the basic Q-Learning for this system; the more advanced DQN approach is then exploited and we show that it successfully maintains the pendulum at the target position in both experiments and simulations. Finally, we explore in the virtual environment the influence of different system’s physical parameters on the control quality.

### RL environment

Our objective is to maintain the pendulum at the target position *θ* = *π* while centering the cart (*x* = 0) at the same time. We perform both experiments and simulations. The system state *s* has been defined with Eq (4). To avoid an angle discontinuity, sin(*θ*) and cos(*θ*) are given to the learning agent instead of only *θ*. The inverted pendulum system is driven by a motor on the cart and it has direct control on the meant cart’s velocity $\dot{x}$ via an applied voltage on the motor. Three actions are offered to the agent at each time step, *i.e*., *a*_*i*_ = (−*U*, 0, + *U*), with *U* ∈ (0, 12V) a fixed voltage. At each time step, the cart can translate in both directions or to keep its current position, according to its dynamics.

Another crucial component for RL is the reward function. The reward is maximum as the objective is reached, *i.e*., the pendulum in its unstable position (*θ* = *π*). In addition, we add the requirement for the cart to be centered around the middle of the track (*x* = 0). For this purpose, there are many options to design the reward function [12], and for simplicity, we have chosen:

$$\begin{array}{c} r\left(\theta ,x\right)=\left(1/2\right)(1-\;\text{cos}\;(\theta ))-{\left(x/x_{0}\right)}^{2}, \end{array}$$ (11)

where *x*_0_ < *x*_*max*_. This additional discardconstraint does not prevent the agent to reach the control objective on the angle. The maximum of this function is equal to one, as *θ* = *π* and *x* = 0.

The normalized return of an episode is computed as the cumulative reward of the entire episode divided by the maximum episode length, *i.e*. 800. Such a definition gives an evaluation of the policy: the closer to 1 the normalized return, the better the episode. An episode is interrupted when the state *s*_*i*_ meets at least one of the following conditions:

1. the dimensionless cart’s position exceeds the physical boundaries, *i.e*., |*x*|>*x*_*max*_; In this case, the agent is strongly penalized and the cumulative reward of the episode is reduced by -400.
2. the angular speed exceed 14 rad/s, since in practice, we would like to avoid the pendulum spinning too rapidly. This value has been chosen according to the mechanical limit of our experimental system.
3. the maximum duration *T*_*ep*_ = 800Δ*t* is attained, where Δ*t* = 0.05 s. This choice has been set to diversify the experience and avoid being stuck in local minimums, which corresponds to roughly 2 or 3 times optimal swing-up time. These values are indeed adapted for an acceptable control quality. In the real experiment, one episode takes approximately 40 s.

At the beginning of every episode, we initialize the system with the cart and the pendulum at rest, *i.e*., *θ* = 0 and *x* = 0. Between two episodes, the system waits 120 s to ensure that the condition $\theta =\dot{\theta }=0$ is satisfied. The learning process consists in accumulating statistics during successive episodes. Plotting the normalized return as a function of the episode number can be noisy and we smooth the data by performing a moving average in Q-learning and DQN over 300 and 30 episodes, respectively, as the former is less stable. Finally, we prefer to represent the learning curve by plotting the normalized return as a function of the total number of time steps to give insights about the true time of the learning process, because some episodes might not run to the end.

#### Experimental setup and methods

**Fig 3:**
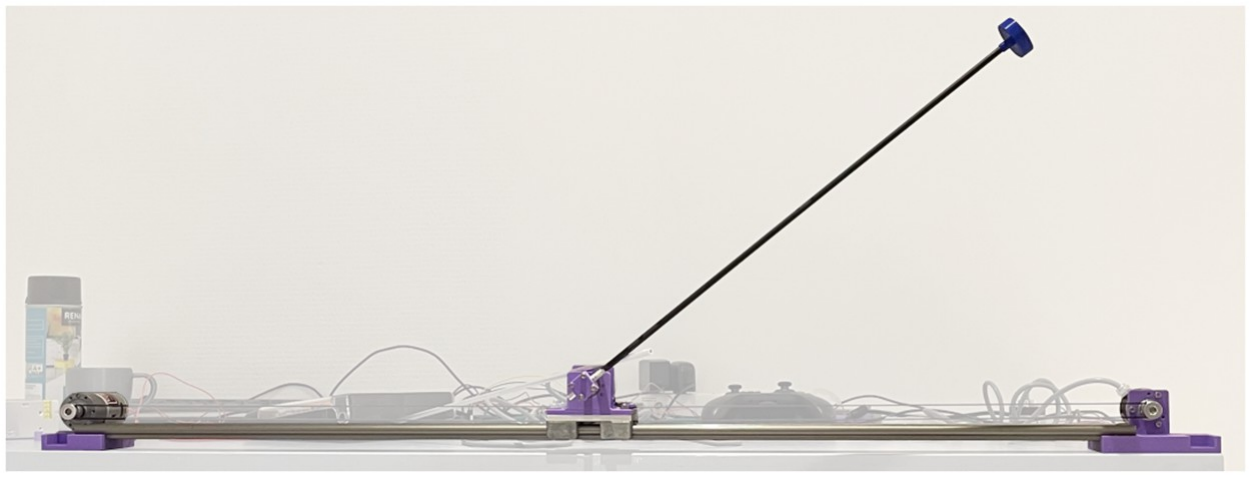
The experimental setup.

The experimental realization of the pendulum is shown in Fig 3. It features a DC motor (model: MFA 970D 12V) which can apply a horizontal force to the sliding base thanks to a transmission belt. An incremental encoder measures the position *x* of the base on a linear track, assuming that *x* = 0 m corresponds to the centered position. The finite length of the track gives the constraint |*x*|<*x*_*max*_, with *x*_*max*_ = 0.35 m. A second encoder mounted on the moving base assesses the angle *θ*. Both are incremental encoders (model: LD3806–600BM-G5–24C) with two phases in quadrature, for a total of 2400 steps per revolution. A Raspberry Pi 4 is used to handle the electronic devices and control the system. It runs a C++ executable, namely the *low-level interface* (LLI), which is responsible of handling the different hardware components and expose the current state of the pendulum to client control applications (see S1 File). The algorithm running on the Raspberry Pi then commands the motor, within the three possible actions. All the code to control the pendulum is open source and available, as well as a reference manual [23].

The exact procedures to measure the physical parameters that appear in Eqs (1) to (3) are described in S2. Their values are summarized in S1 File.

#### Simulations

In the experimental setup, the state information is gathered directly from the physical world, and the agent interacts with the environment via the LLI. In the virtual setup, the agent’s state is updated through Eqs (1) to (3). Details can be found in our code [23]. The effects of the voltage *U*, the dry friction acting on the motorized base *f*_*c*_ and the viscous friction *k*_*v*_ of the pendulum were investigated systematically in simulation. The same holds for the influence of the noise amplitude *σ*_*θ*_ and $\sigma_{\dot{\theta }}$ on the control quality: we have introduced a Gaussian noise to the measurement of the pendulum angle *θ*, *i.e*., at each instant *t* = *i*Δ*t*, $\theta_{i}∼N\left(\theta_{i}^{m},\;\sigma_{\theta }^{2}\right)$, where N refers to the normal distribution, $\theta_{i}^{m}=\theta_{i-1}+\dot{\theta }\Delta t$ is updated from the previous state. Naturally, a noise of amplitude $\sigma_{\dot{\theta }}=\sigma_{\theta }/\Delta t$ was then introduced to the $\dot{\theta }$ measurement.

### Q-learning

In Q-learning, the observation space $\left(\text{sin}\left(\theta \right),\text{cos}\left(\theta \right),\dot{\theta },x,\dot{x}\right)$ is discretized into different number of bins, whose sizes is matter of compromise. A Q-table with low resolution results in relatively fast simulations and limits the use of computer memory. On the other hand, the resolution needs to be high enough to ensure the success of the learning process. As an example we start with a sparse and homogeneous discretization with nBins = (10, 10, 10, 10, 10). In this case we expect the Q-table to contain 3 ⋅ 10^5^ elements, given that there are three possible actions.

The Q-table size gives a minimal estimate of the total number of time steps to learn assuming that the agent needs to visit each element of the table. This number is 10–100 times higher in practice given that the basic Q-learning algorithm usually suffers a low sample-efficiency [12]: some elements are never evaluated while some others can be updated regularly.

**Fig 4:**
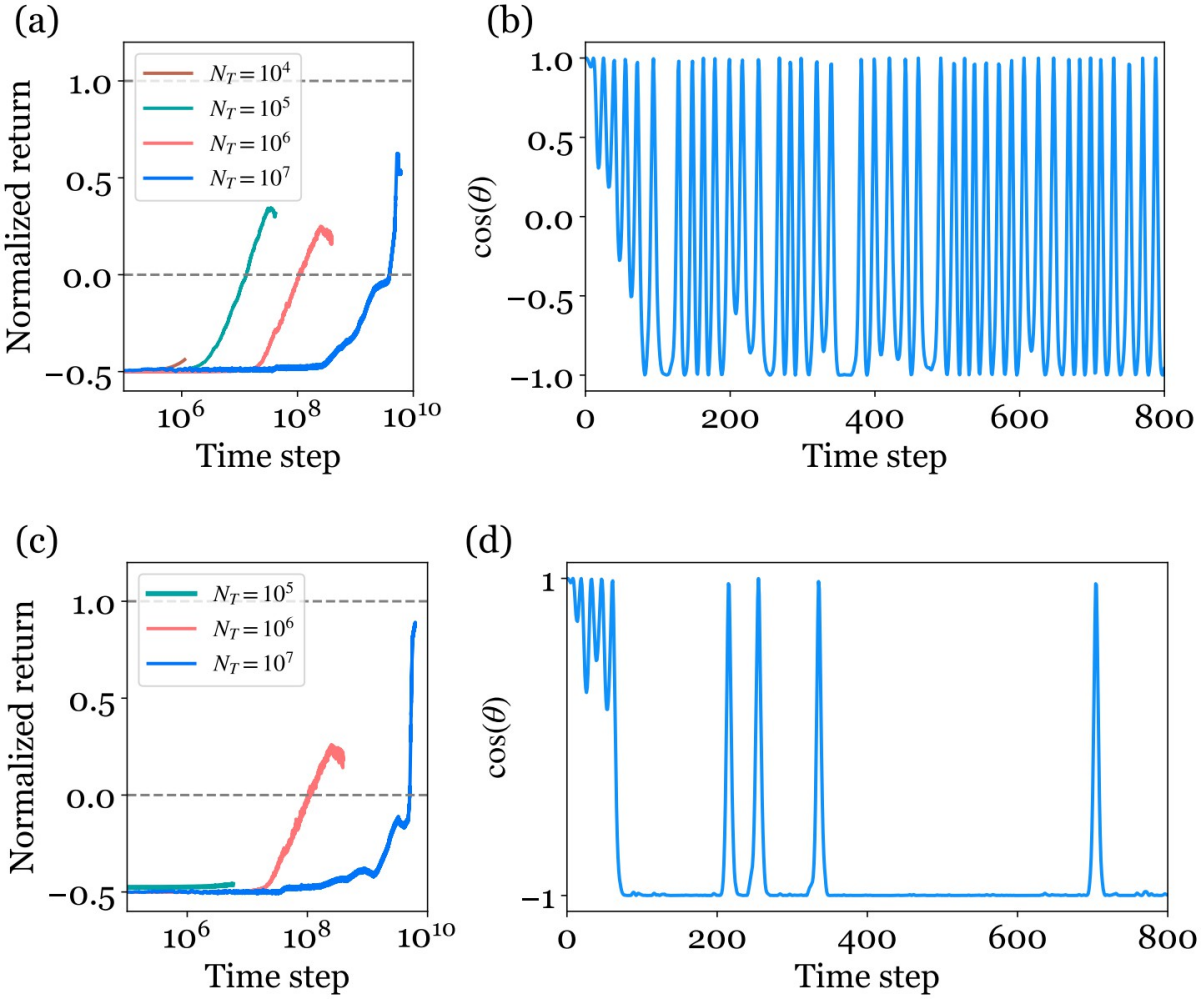
Learning results using the basic tabular Q-learning implementation. Left: Normalized return as a function of the number of time steps for different total number of episodes *N*_*T*_. Right: Temporal evolution of cos *θ* in the best episode of the longest learning process (*N*_*T*_ = 10^7^). The observation space $\left(\text{sin}\left(\theta \right),\text{cos}\left(\theta \right),\dot{\theta },x,\dot{x}\right)$ is discretized homogeneously into different number of bins: a) and b) nBins = (10, 10, 10, 10, 10), c) and d) nBins = (50, 50, 50, 10, 10).

We have tested the Q-learning approach in simulation with different total number of episodes *N*_*T*_ from 10^4^ to 10^7^. We recall that one episode contains 800 time steps at maximum; the average number of time steps per episode is lower in practice due to numerous interrupted episodes at the beginning of the learning process. The technical details such as the value of the hyperparameters are found in S4. Given the expression of the reward function and of the penalty, the cumulative reward spans from -0.5 (the cart goes quickly out of the track) to 1 (successful learning). Below 10^6^ time steps, the normalized return remains close to its minimum. The system requires at least 10^7^ time steps (10^5^ episodes) to observe an increase of the normalized return above 0 (Fig 4a). Even in this case, the cumulative reward remains low, around 0.3, and reaches 0.55 at most as the number of time steps is increased to 10^10^. For such an episode, the pendulum can be maintained at its vertical position only in a short amount of time, otherwise the pendulum oscillates (Fig 4b). Transposed to experiments with a physical time interval Δ*t* = 0.05s, 10^7^ time steps correspond to 6 days of experiments!

We can nevertheless discuss the effect of the discretization of the Q-table, which is too low in the former example to reach a cumulative reward close to 1 even after a very large number of time steps. In the following, we estimate the typical value *n*_*θ*_ for the bin in *θ*: the discretization interval is Δ*θ* = 2*π*/*n*_*θ*_. In order to ensure the learning objective, the time interval separating two actions must not be too large with respect to this discretization. We expect that Δ*t* should be smaller than the typical time the agent lasts in one interval: we can assess this duration in the limit of small damping. By assuming that the pendulum is weekly damped, we approximate the Eq (1) with $\ddot{\theta }+\omega^{2}\;\text{sin}\;\theta =0$. Consequently we write the energy conservation $\frac{1}{2}{\dot{\theta }}^{2}\left(t\right)=\omega^{2}(\text{cos}\;\theta (t)-\text{cos}\;\theta (0))$, where $\dot{\theta }\left(0\right)=0$. Between two iterations the angle varies within an increment Δ*θ* and we write *θ*(*t*) = *θ*(0) + Δ*θ*, Δ*θ* ≪ 1:

$$\text{cos}\;\theta (t)=\text{cos}\left(\theta \left(0\right)\right)-\Delta \theta \;\text{sin}\;\theta \left(0\right)+o{\left(\Delta \theta \right)}^{2}$$ (12)

$$\begin{array}{cc} \dot{\theta } & =\frac{\Delta \theta }{\Delta t}, \end{array}$$ (13)

such that we deduce that:

$$\begin{array}{c} n_{\theta }=\frac{\pi }{\omega^{2}\Delta t^{2}}\frac{1}{\text{sin}\;\theta (0)}. \end{array}$$ (14)

This gives the order of magnitude *n*_*θ*_ ∼ 50. The presence of a divergence near the unstable equilibrium shows that the discretization must be refined at least near cos(*θ*) = −1.

Consequently, we tested a finer resolution nBins = (50, 50, 50, 10, 10) with sin(*θ*), cos(*θ*) and $\dot{\theta }$ discretized into 50 bins. The computation memory increases exponentially with the size of the Q-table and any finer resolution would be unpractical. As observed in Fig 4c, it takes at least 10^8^ time steps to see a normalized return above 0. After about 5.6 × 10^9^ time steps (7 × 10^6^ episodes), the system has finally learned reasonably well and obtain a normalized return of ∼0.8: the pendulum can stay in the goal position for a finite period, but quickly falls over to be quickly swung back up again (Fig 4d).

The inefficiency of the learning is rationalized by the fact that the matrix representation of Q-Table is not adapted to solve the swing-up problem. To update the action-value function more efficiently, a better function approximation is needed. In that regard, artificial neural networks show very promising capabilities and is data efficient [10].

### Deep Q learning

In this section, we implement the Deep Q Learning technique. In this approach, the Q-Table for approximating the Q-function is replaced by an Artificial Neural Network (ANN), which is named Deep Q Network (DQN). We have shortly introduced ANN in S3. For our purposes, we use for the ANN a dense neural network architecture with 2 hidden layers having 256 nodes each. Five nodes receive the values of the state *s*, and three terminal nodes give the Q-value for each of the 3 actions. Similar to any other deep learning algorithm, the training of DQN depends on the hyperparameters, which determine the network policy structure, the learning strategy and the learning speed. We offer a set of fixed hyperparameters (see S1 File), which is robust for our system.

In parallel to experiments, we performed simulations of the model. For both approaches, the features and quality of the learning process are evaluated.

Note that the maximal number of time steps (150000) for the complete training is chosen so that the steady state average value is reached in both real and virtual experiments.

We evaluate the policy performance every 5000 time steps with an inference. It consists in testing a greedy policy during one complete episode, with the initial condition $\left(\theta ,\dot{\theta },x,\dot{x}\right)=\left(0,0,q0,0\right)$. This protocol is applied directly in experiments, while in simulations, the inference curve consists in computing the evolution of the average normalized return of 10 episodes (instead of only one in experiments) with equidistant initial conditions: (*θ*_0_ ∈ (−10°, 10°) and $\left(\dot{\theta },x,\dot{x}\right)=\left(0,0,0\right)$). This allows to test the robustness of the policy in simulation, *i.e*., the capacity to generalize and achieve a high normalized return from different initial states, other than the particular initial state of the learning process. This protocol however is not viable in experiments since in practice it is difficult to control precisely the initial angle of the pendulum other than its equilibrium position. Finally, the best learned policy in the sequel corresponds to the DQN model that obtained the highest normalized return among all inferences.

#### Experimental results

**Fig 5:**
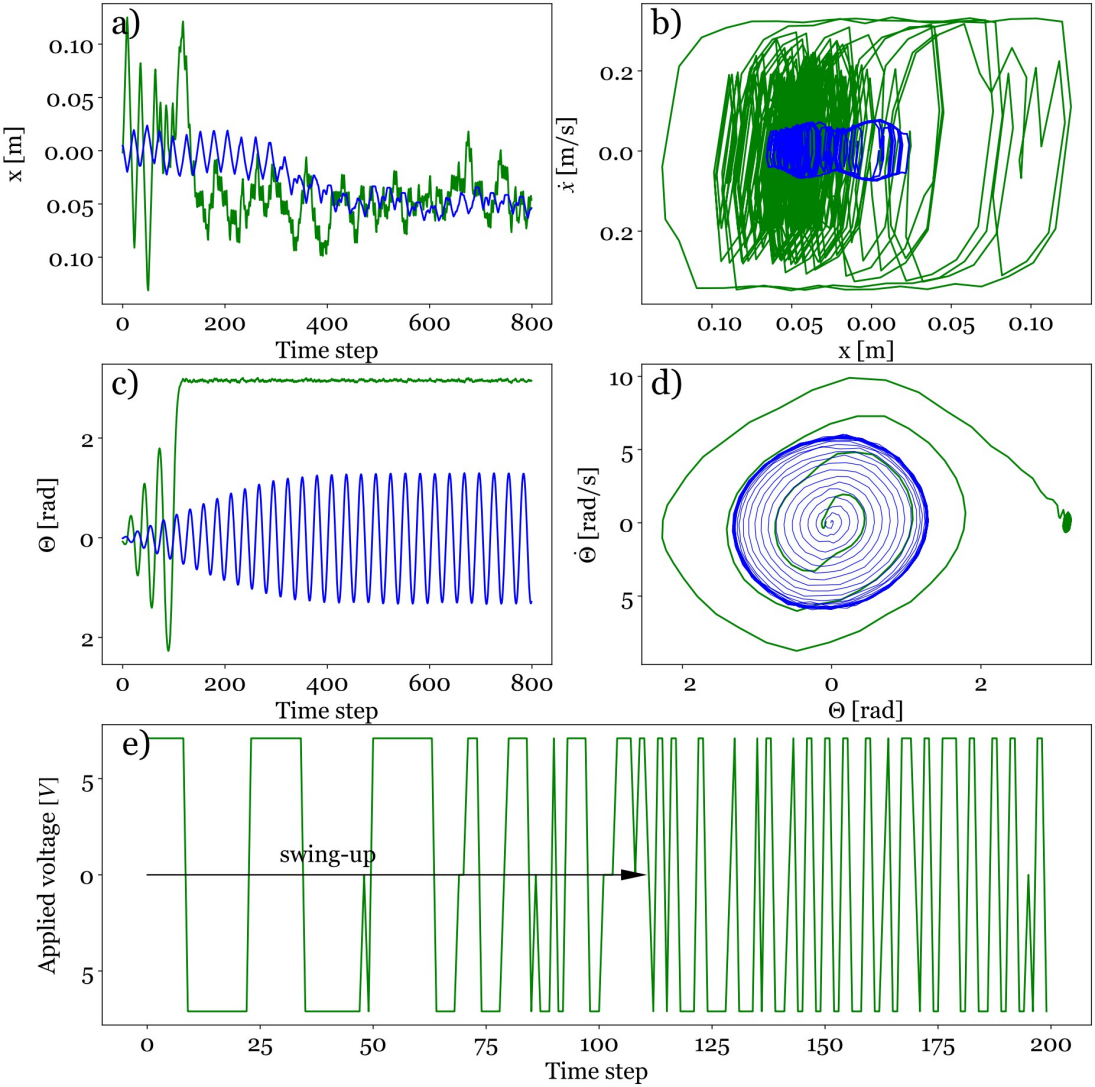
Experimental results with the best policies in inference for two different applied voltages *U* = 2.4 V (blue) and 7.1 V (green): a) Temporal evolution of the cart’s position *x* during one episode b) trajectory of the cart in the $\left(x,\dot{x}\right)$ space c) Temporal evolution of the pendulum’s angle *θ* during one episode d) Trajectory of the pendulum in the $\left(\theta ,\dot{\theta }\right)$ space e) Temporal evolution of the applied voltage during the first 200 time steps.

We first discuss the results of the outlined DQN algorithm obtained with the experimental setup. The only control parameter is the applied voltage *U*, which is directly proportional to the target cart’s velocity value ${\dot{x}}_{c}$ Eq (3). Fig 5 displays the temporal evolutions (a) of the cart’s position and (b) of the pendulum’s angle during a single episode for the best learned policies. Two distinct voltages were tested: *U* = 2.4 V and *U* = 7.1 V: we illustrate the corresponding learning processes in a movie (See S1 Video).

The voltage *U* = 2.4 V is not sufficient to swing up the pendulum, and the best policy yields an oscillation of the pendulum around 0. This means that the energy provided with this voltage is not high enough to swing up the pendulum or that the total duration of one episode, 800 time steps, is not large enough to increase the maximal angle, period after period. Given that the maximum angle is reached after 300 times steps already, the first assumption is probably the good one.

**Fig 6:**
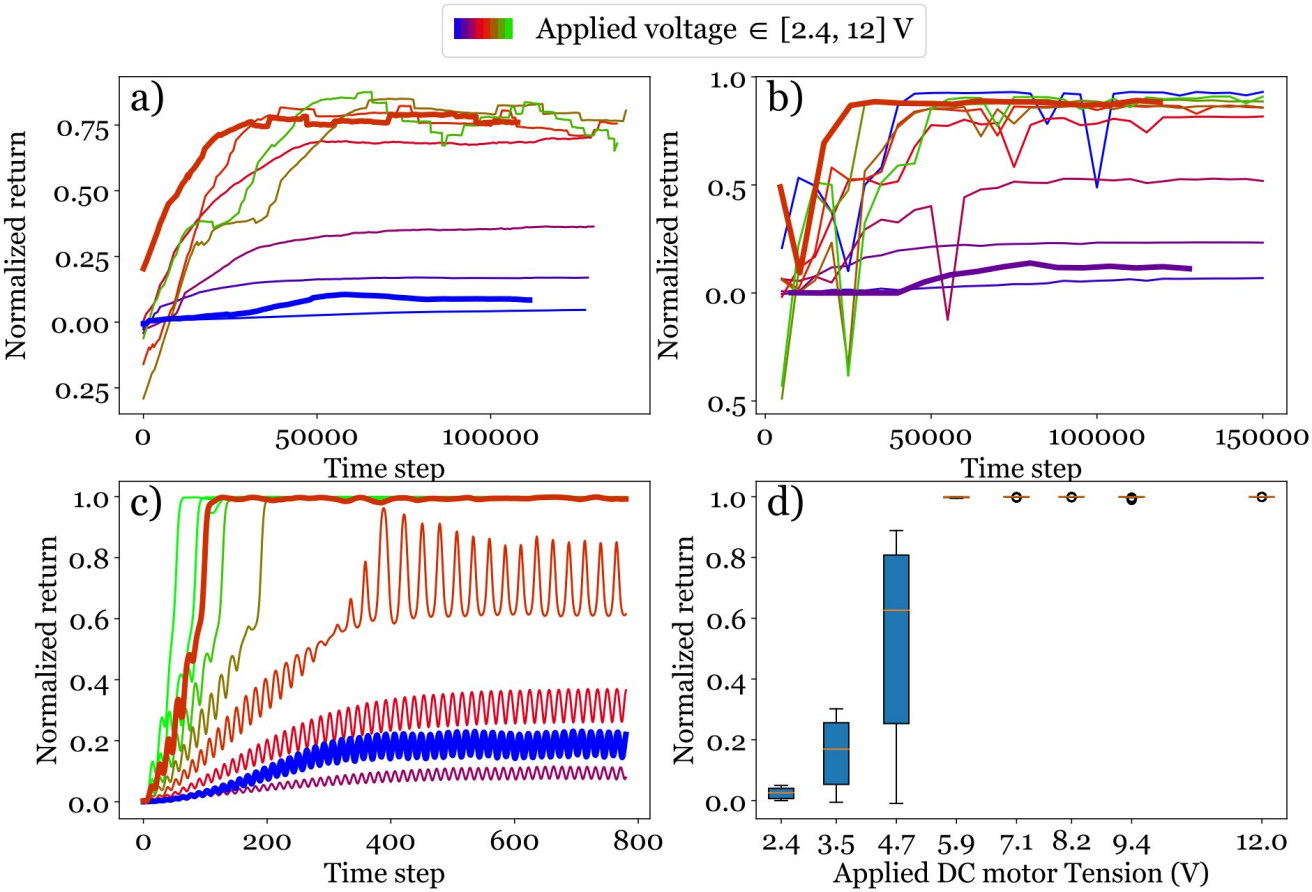
Influence of the applied voltage on the learning process. Thin curves correspond to different simulations, while thick curves refer to the experimental observations. a) Learning curve. b) Inference curve built from inferences performed every 5000 time steps. c) Temporal evolution of the reward for the episode initiated at *θ*_0_ = 0 following the best learned policy. d) Statistics over 10 episodes initiated with *θ*_0_ between −10° and 10° of the plateau reward following the best learned policy.

For the other voltage *U* = 7.1 V, the cart initially oscillates with a large amplitude and the pendulum swings up after about the equivalent of almost 3 periods. As soon as the unstable equilibrium is reached, the cart turns into a vibration regime with smaller amplitude to maintain the pendulum balanced upward around *θ* = *π*. The learning and the inference curves (see Fig 6, thick solid lines) reveal exactly the same results that for *U* = 7.1 V, the normalized return in both learning and inference reaches a high plateau value of ∼0.8 − 0.9, indicating a successful control, while for *U* = 2.4 V, the normalized return stays very low around 0.1.

#### Simulation results

In this section, we perform simulations and test different important physical parameters which could influence the control quality. All the parameters are kept constant and defined with the Table 1 and 2 of the SI except the one investigated. The voltage is set to *U* = 12 V and the noise $\sigma_{\theta }=\sigma_{\dot{\theta }}=0$ if not specified.

#### Effect of action amplitude—Applied voltage on the DC motor

We have shown with our experimental setup that the action amplitude plays a crucial role in the task: a low voltage applied on the DC motor results in a failure of control. Here we test a range of *U* from 2.4 to 12 V in the virtual environment and the results are presented in Fig 6. First, we note that the simulation results are consistent with those found in experiments (thick curves), *i.e*., both normal and thick curves of (*U* = 7.1 V) as well as (*U* = 2.4 V) show similar trend. Fig 6a displays the learning curves. The normalized return increases and then reaches a plateau for all the applied voltages. However, up to *U* = 4.7 V, the plateau value is smaller than 0.4, close to that observed using Q-learning algorithm, referring to an oscillation around the stable position. Above 4.7 V, DQN algorithm gives satisfying performance during the learning process.

To assess the performance of the optimal policy obtained for each applied voltage, we plot the inference results in Fig 6b. Because there is no exploration and the optimal action is chosen at each time step, the plateau value of each inference curve is expected to be greater than the corresponding learning curve. Nevertheless, some inferences exhibit negative peaks associated to the fact that among the 10 episodes that are averaged to measure the normalized return of an inference, some of them are terminated by the cart reaching *x*_*max*_ and are strongly penalized consequently. These negative peaks disappear as the number of time steps increases and the learning process continues. A normalized return between 0.8–0.9 is a good value as it is calculated from the averaging on one episode, and this includes the initial stage before swing up. This can be seen in Fig 6c where the learning process is probed by plotting the time evolution of the reward for an episode initiated at *θ*_0_ = 0 following the best learned policy obtained after the 150000 time steps. From *U* = 5.9 V, the plateau of the reward is around 1 and the system reaches the objective. This figure also reveals that the higher the applied voltage is, the quicker the swing-up is achieved. To probe the robustness of the best learned policy for each applied voltage, we have measured the average of the plateau reward for 10 episodes initiated with equidistant initial values of *θ*_0_ between −10° and 10°. Statistics over these 10 episodes are represented by a box-plot of the reward as a function of *U* (Fig 6d). It shows that the pendulum can operate and maintain a swing up for some values of *θ*_0_ even for *U* = 4.7V, but that this behavior becomes robust only for *U* ≥ 5.9V.

#### Effect of the physical parameters

In what follows, we numerically investigate the robustness of the learning process with respect to the two frictions coefficients and to the two sources of noise.

**Fig 7:**
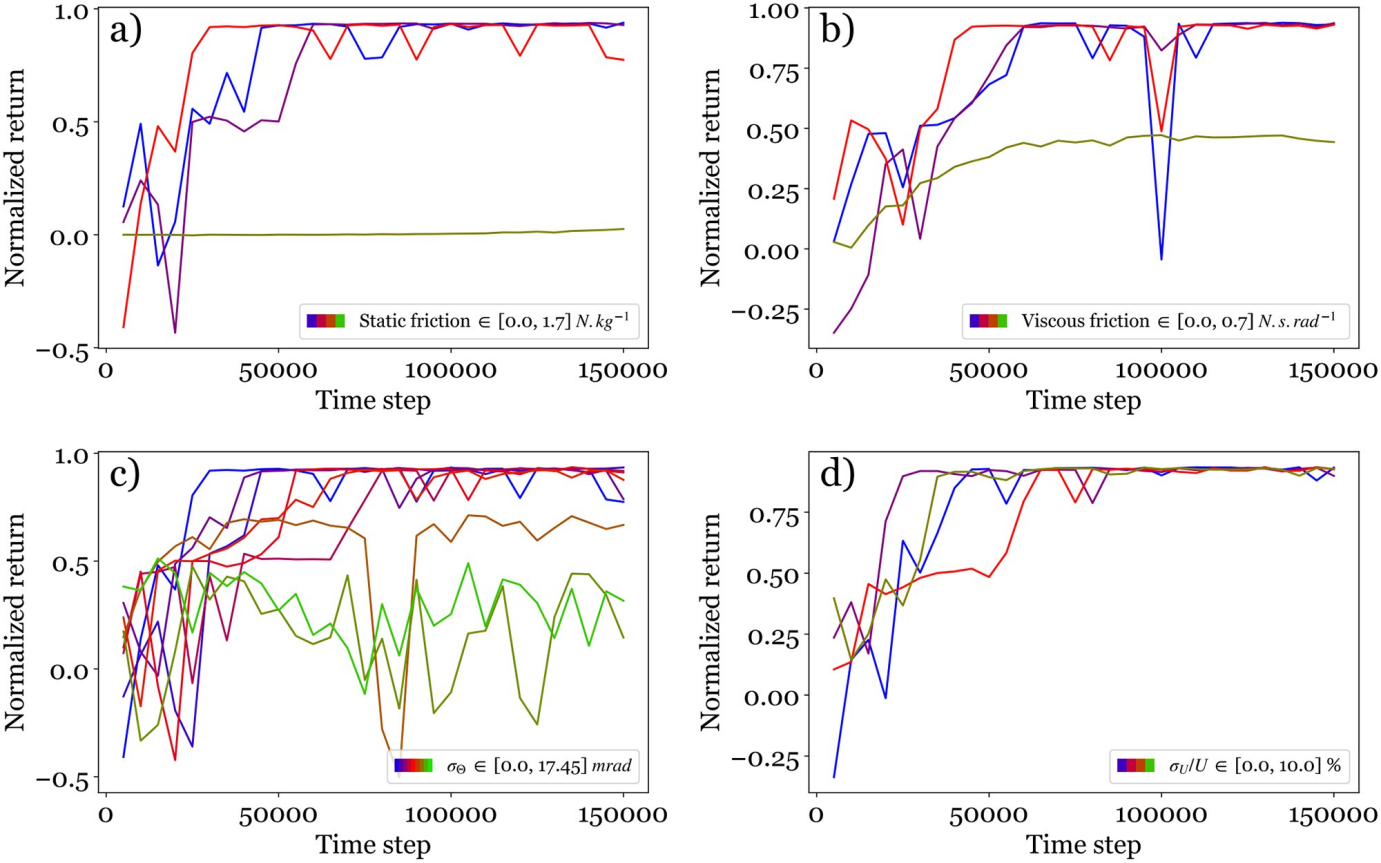
Influence of the physical parameters on the control: Inference curves of a) static friction, b) viscous friction, c) measurement noise and d) action noise.

In Fig 7a, the static friction is varied from 0 to 11.7 N.kg^−1^, keeping the other parameters constant. We observe that the value 1.17 N.kg^−1^ measured with the real system does not perturb the learning process in comparison to a system without friction. However, increasing tenfold this parameter value prevents the system from learning correctly. In Fig 7b, the viscous friction is varied from 0 to 0.70 N.kg^−1^. Again the experimental value 0.07 N.s.rad^−1^ exhibits a good learning performance but multiplying this value by 10 would prevent the agent to drive the pendulum to the target.

As mentioned in the experimental setup description, the real system has uncertainties associated to the measurement of the angle *θ*. In the virtual environment, this is accounted for by Gaussian noises of standard deviations *σ*_*θ*_ and *σ*_*θ*_/Δ*t* for the measurements of *θ* and $\dot{\theta }$ respectively. From the real system, we have evaluated *σ*_*θ*_ ∼ 2.6 mrad. Here we probe values ranging between 0 and 175 mrad in simulation (Fig 7c). Clearly, low measurement noises, *i.e*., *σ*_*θ*_ < 8.7 mrad, result in a perfect control quality as observed with high plateau values of the inference curves. A noise amplitude of 17.5 mrad is still acceptable. Beyond this value, the pendulum can’t be driven to its unstable position.

Finally, we examine the effect of an associated degree of uncertainty on the command sent to the motor, thus a Gaussian noise of standard deviation *σ*_*U*_ is added to the voltage *U* in simulation. We show in Fig 7d that, up to a noise level of *σ*_*U*_/*U* ≃ 0.1, a good control is achieved. This condition is not restrictive and is easily obtained with classical systems. A moderate noise does not seem to impact the quality of the learning process.

## Conclusion

In this article, we have revisited in a pedagogical context, the stabilization of an inverted pendulum, a classical problem in dynamics and control theory. We first recalled the physical model of such a system and the control objective. Two model-free Reinforcement Learning algorithms were investigated both in experiments and in simulations, which offers an accurate description of real experiments. In terms of the control quality, the basic Q-Learning method is found not efficient while the more advanced algorithm DQN successfully accomplishes the stabilization of the pendulum in its unstable position, independently of the initial condition. Finally, we studied the influence of some extensive physical parameters on the control quality in simulation with the virtual environment. The robustness of the DQN approach has been therefore validated, both in terms of parameter range, but also in terms of initial conditions: the RL always drives the pendulum in its unstable position, independently of the initial state. An admissible range of physical parameters were determined, which can be used to guide the elaboration of experimental setups.

Meanwhile, we deliberately chose to use discrete actions for simplicity, but there exists many other RL algorithms which can work with continuous action spaces, for instance the Soft Actor-Critic (SAC) algorithm [26]. Using continuous action space unquestionably enables a finer control, but it would take more resources and time to train the RL model due to additional complexity, and is less suitable for the scope of this article.

For public outreach, we provide all the details in an open-source code repository.

## Supporting information

S1 File Supplementary material to the manuscript. (PDF)

S1 Video The learning process and the quality of the control for the pendulum. (MP4)
