# Supplementary material for: Reinforcement learning approach to control an inverted pendulum: A general framework for educational purposes
Source: PLoS One. 2023 Feb 13;18(2):e0280071. doi: 10.1371/journal.pone.0280071 (PMC9925229; doi:10.1371/journal.pone.0280071)
Supplement: S1 File — (PDF) [file pone.0280071.s001.pdf]

Supporting Information to: Reinforcement learning approach to control an inverted pendulum: a general framework for educational purposes.

Sardor Israilov[1,2], Li Fu[1], Jesús Sánchez-Rodríguez[1,3], Franco Fusco[2], Guillaume Allibert[2], Christophe Raufaste [1,4], Médéric Argentina [1,*]

1 Université Côte d'Azur, CNRS, INPHYNI, France
2 Université Côte d'Azur, CNRS, I3S, France
3 Laboratory of Fluid Mechanics and Instabilities, École Polytechnique Fédérale de Lausanne, CH-1015 Lausanne, Switzerland
4 Institut Universitaire de France (IUF), France

All the authors contributed equally to this work.
* mederic.argentina@univ-cotedazur.fr

Low-level Interface (LLI) At each major control cycle, the LLI processes the raw measurements from the encoders by smoothing them with a digital 4th-order Butterworth filter [28] and by differentiating them numerically in order to estimate $\dot{x}$ and $\dot{\theta}$. For the communication, we use the ZeroMQ[1] library. This allows to write client controller applications that do not need to focus on the low-level management of hardware resources. In addition, clients can be run either from the Raspberry Pi 4 or from any other machine that is able to connect to the board, *e.g.*, via the local network or via WiFi. This opens the possibility to write client applications in potentially any programming language supported by ZeroMQ. Our client applications are written in Python and C++.

Measurements of the physical parameters The values of the physical parameters of the cart-pole are displayed in Table 1. The pendulum mass was measured with a scale. The natural frequency ω and viscous friction coefficient k_v were inferred from the signal $\theta(t)$ of the free oscillations of the pendulum with a blocked cart as expected by Eq. 1 of the main text with $\ddot{x} = 0$. We show in fig. 1a, the relaxation dynamics of the pendulum, as well as the result of the numerical prediction of the model with the best fitted parameters. The parameters τ, f_c, f_d and k_U in Eqs. (2-3) (of the main text) are inferred by imposing step functions as voltages and measuring the cart velocity as a function of time. Again, parameters are deduced by the best interpolations (Fig. 1b). In Fig. 1c, we observe in more details the effect of the three parameters f_c, f_d and k_U on the discontinuity on the velocity-axis, the up-down asymmetry and the slope respectively, while plotting the steady state velocity as a function of the applied voltage. The uncertainty on the angular velocity $\dot{\theta}$ is correlated to σ_θ and to the time resolution $\Delta t \simeq 0.05\,\mathrm{s}$. This gives an uncertainty $\sigma_{\dot{\theta}} = \sigma_\theta/\Delta t \simeq 52\,\mathrm{mrad\,s^{-1}}$.

Methodology for training RL agents All the simulations and experiments were driven by a Dell Precision 7550 using its internal GPU. For one simulation with 15 10^5 time steps with logging and evaluation loops, it takes 10.7 minutes using GPU (NVidia Quadro T2000), and 13.43 minutes using CPU only (Intel(R) Core(TM) i7-10875H CPU @ 2.30GHz).

[1]ZeroMQ: `https://zeromq.org/` (accessed on July 23rd, 2021).

Name	Value
Mass of the pendulum (m)	$0.075\,\mathrm{kg}$
Natural frequency of the pendulum (ω)	$4.882\,\mathrm{rad\,s^{-1}}$
Viscous friction coefficient of the pendulum (k_v)	$0.07\,\mathrm{N\,s\,rad^{-1}}$
Electro-mechanical time constant(τ)	$0.0482\,\mathrm{s}$
Static gain of the motor (k_U)	$0.051\,\mathrm{m\,s^{-1}\,V^{-1}}$
Static friction coefficient of the cart per unit mass (f_c)	$1.166\,\mathrm{N\,kg^{-1}}$
Static offset per unit mass (f_d)	$-0.097\,\mathrm{m\,s^{-2}}$

Table 1. Measured physical parameters.

1. **Artificial Neural Networks**
   Artificial Neural Networks (ANN) are an assembly of idealized biological neurons [12]. Each neuron, labelled k, possesses a state S_k and receives a signal p_k from other neurons. This incoming information writes:

   $$p_k = \sum_j S_j w_{jk} + w_{0k},$$

   where w_{jk} measures the weight of the link between the neurons j and k. In general, there is also a bias w_{0k} for each neuron. The incoming signal p_k is treated through a function f to define the new state of the neuron:

   $$S_k \leftarrow f\left(p_k\right),$$

   where f is the activation function.

   In our problem, the ANN's input layer have 5 neurons that handle the five components of the observation $(\sin(\theta), \cos(\theta), \dot{\theta}, x, \dot{x})$. Each of these five neurons is connected to the first hidden layer consisting of 256 nodes, which are also connected to a second hidden layer of also 256 nodes. For the two hidden layers, we use the Rectified Linear Unit function (ReLU) [12, 29]:

   $$f(x) = \max(0, x).$$

   The network's output layer is made up of 3 neurons, which gather information from the previous hidden layer. Each output neuron represents the action-value of 3 possible actions for the current state. The training process updates the unknown parameters w_{jk} and w_{0k}, in order to minimize the error between the output of the ANN, and the estimated true value based on the real reward given by the environment.

2. **Additional techniques**
   In addition, to stabilize the learning process and obtain more reliable results, DQN also employs a number of additional techniques such as replay buffer, fixed Q-targets [7] and gradient clipping which improves the stability of learning by clipping the TD error eq. (2) to [-1,1] interval. In our configuration, the learning process happens at the end of every episode through the use of gradient descent applied on mini-batches of transitions (s_i, a_i, r_i, s_{i+1}) sampled from a buffer composed by 50000 (state/action/reward/next state) values.

   This is the concept of an experience replay. The replay buffer permits to reuse the transitions in an update, and also to break temporal correlation of those

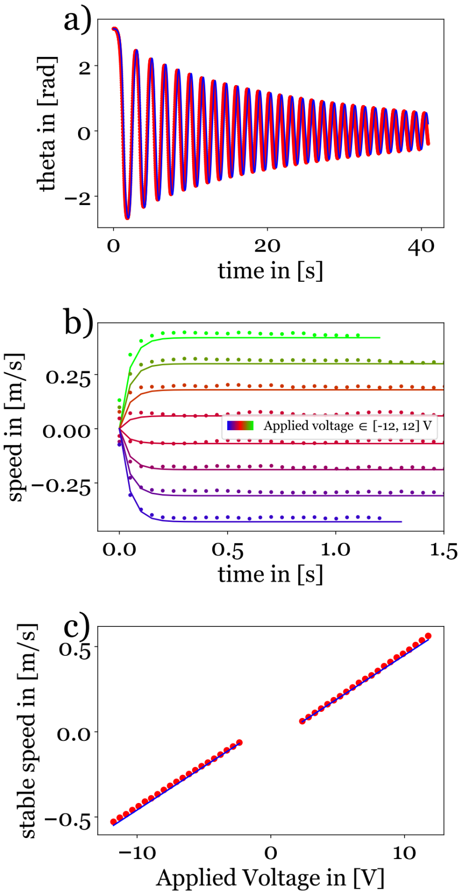

Fig 1. Determination of the physical parameters from interpolations (solid blue curves) of experimental data (red dots). a) Angle evolution of a free oscillation of the pendulum. b) Cart's velocity evolution with different voltages. After a short transition period, the cart's velocity reaches a plateau for all the voltages. c) Linear dependence of the plateau values on the applied voltages.

transitions for the learning process by shuffling the data. The weight updates of the neural network follows the steepest gradient scheme:

$$\Delta(w_{jk}) = \alpha \left(\underbrace{R + \gamma \max_a Q(s_{i+1}, a_{i+1}, w_{jk}^-)}_{\text{Target}} \right. \tag{1}$$

$$\left. - \underbrace{Q(s_i, a_i, w_{jk})}_{\text{Local network value}} \right) \nabla Q(s_i, a_i, w_{jk}), \tag{2}$$

where w_{jk} refers to the local network parameters, w_{jk}^- refers to the target network parameters. The TD target approximates the true $Q(s_i, a_i)$ in Eq. (10) of the main text, and the update is done proportionally to the error between the approximated true action-value function and the current value. To avoid the the target value that changes frequently over time, the fixed network was introduced,

thus decoupling the target value from the weight update. This increased robustness and stability of the learning. After 1000 time steps (table 2), the target network parameters w_{jk}^{-} are updated with the local network parameters w_{jk} [29].

	Q-learning	DQN
Learning rate (α)	0.01	0.0003
Exploration ratio (ϵ)	ϵ varies following Eq. 3 and $\epsilon_{min} = 0.1$	0.178
Discount factor (γ)	0.99	0.995
Decay factor (d)	$10^3 - 10^6$	N/A
Buffer size	N/A	50000
Batch size	N/A	1024
Network architecture	N/A	2 hidden layers with 256 neurons
ANN optimizer	N/A	Adam [30] with default parameters
Loss type	N/A	Huber
Activation function	N/A	Rectified Linear Unit (ReLU)
Target update interval (C)	N/A	1000
Train frequency	1 step	1 episode
Gradient steps	N/A	as many as there were steps since last neural net update

Table 2. Hyperparameters for Q-learning and DQN.

Hyperparameters for RL

Q-learning The hyperparameters for Q-learning were set as follows. We set $\alpha = 0.01$ in Eq. (10) of the main text. As for the hyperparameter ϵ (ϵ-greedy policy), it is a good practice to promote the exploration in the early stage of the learning process with ϵ close to 1, while a small ϵ helps to converge quickly at the end of the process. Here ϵ decreases as a function of time:

$$\epsilon = \max(\epsilon_{min}, \min(1, 1 - \log_{10}((n + 1)/d)) \tag{3}$$

, where n is the number of current episode. The decay coefficients d and ϵ_{min} are hyperparameters that can be tuned ; in this work we took $d = N_T/10$ and $\epsilon_{min} = 0.1$.

DQN The parameters were tuned with the help of Optuna [31] framework. We discovered that the most sensible hyperparameters are network architecture, batch size and exploration rate. The complete tuning focused on the following parameters:

- **Buffer size**: the size of a buffer with transitions $(\cos(\theta), \sin(\theta), \dot{\theta}, x, \dot{x})$ used for learning the weights of the policy.
- **Batch size**: number of samples used for the gradient descent update of neural network. In practice it should be large enough to avoid biased experience, but not too large to slow the learning.
- **Learning rate**: an extent at which we update the "state-value" function at each step.

- **Gamma**: discount rate of future steps, which tells how the present is more valuable than the future.
- **Exploration rate**: the rate at which an agent explores (acts randomly) in the environment
- **Network size**: size of the dense neural network for $Q(s,a)$ function approximation
- **Target update interval**: it is the interval after which we update the target. In general, the bigger the value, the more stable is the training, but decreases the learning speed
- **Train frequency**: the frequency of learning the weights from the experience; in our case we train the neural network at the end of every episode, since it is the most suitable way to be applied on real-life robotic reinforcement learning

References

1. Lundberg KH, Barton TW. History of Inverted-Pendulum Systems. IFAC Proceedings Volumes. 2010 Jan;42(24):131–135.
2. Boubaker O. The inverted pendulum benchmark in nonlinear control theory: a survey. International Journal of Advanced Robotic Systems. 2013;10(5):233.
3. Sugihara T, Nakamura Y, Inoue H. Real-time humanoid motion generation through ZMP manipulation based on inverted pendulum control. In: IEEE International Conference on Robotics and Automation. vol. 2; 2002. p. 1404–1409.
4. Lee GH, Jung S. Design and control of an inverted pendulum system for intelligent mechatronics system control education. In: IEEE/ASME International Conference on Advanced Intelligent Mechatronics; 2008. p. 1254–1259.
5. Lazarini AZN, de Souza Ribeiro JM, Jorgetto MFC. Low cost implementation of a inverted pendulum control system. In: 11th IEEE/IAS International Conference on Industry Applications; 2014. p. 1–5.
6. Bakaráč P, Kalúz M, Čirka L. Design and development of a low-cost inverted pendulum for control education. In: 21st International Conference on Process Control (PC); 2017. p. 398–403.
7. Mnih V, Kavukcuoglu K, Silver D, Graves A, Antonoglou I, Wierstra D, et al. Playing Atari with Deep Reinforcement Learning. arXiv:13125602. 2013.
8. Silver D, Schrittwieser J, Simonyan K, Antonoglou I, Huang A, Guez A, et al. Mastering the game of Go without human knowledge. Nature. 2017;550:354–359.
9. Buşoniu L, de Bruin T, Tolić D, Kober J, Palunko I. Reinforcement Learning for Control: Performance, Stability, and Deep Approximators. Annual Reviews in Control. 2018;46:8–28.
10. Riedmiller M. Neural Reinforcement Learning to Swing-Up and Balance a Real Pole. In: IEEE International Conference on Systems, Man and Cybernetics; 2005. p. 3191–3196 Vol. 4.
11. Kim H, Jordan M, Sastry S, Ng A. Autonomous Helicopter Flight via Reinforcement Learning. In: Thrun S, Saul L, Schölkopf B, editors. Advances in Neural Information Processing Systems. vol. 16. MIT Press; 2004. .

12. Sutton RS, Barto AG. Reinforcement learning: An Introduction (Second edition). The MIT Press, Second edition; 2012.

13. Huang J, Ding F, Fukuda T, Matsuno T. Modeling and velocity control for a novel narrow vehicle based on mobile wheeled inverted pendulum. IEEE Transactions on Control Systems Technology. 2012;21(5):1607–1617.

14. Sun W, Su SF, Xia J, Wu Y. Adaptive tracking control of wheeled inverted pendulums with periodic disturbances. IEEE Transactions on Cybernetics. 2018;50(5):1867–1876.

15. OpenAI Gym;. `https://https://gym.openai.com/`.

16. Koryakovskiy I, Kudruss M, Babuška R, Caarls W, Kirches C, Mombaur K, et al. Benchmarking model-free and model-based optimal control. Robotics and Autonomous Systems. 2017;92:81–90.

17. Manrique Escobar CA, Pappalardo CM, Guida D. A Parametric Study of a Deep Reinforcement Learning Control System Applied to the Swing-Up Problem of the Cart-Pole. Applied Sciences. 2020;10(24).

18. Zheng Y, Li X, Xu L. Balance control for the first-order inverted pendulum based on the advantage actor-critic algorithm. International Journal of Control, Automation and Systems. 2020;18(12):3093–3100.

19. Surriani A, Wahyunggoro O, Cahyadi AI. Reinforcement Learning for Cart Pole Inverted Pendulum System. In: 2021 IEEE Industrial Electronics and Applications Conference; 2021. p. 297–301.

20. Özalp R, Varol NK, Taşci B, Uçar A. In: Tsihrintzis GA, Jain LC, editors. A Review of Deep Reinforcement Learning Algorithms and Comparative Results on Inverted Pendulum System. Springer International Publishing; 2020. p. 237–256.

21. Kumar S. Balancing a CartPole System with Reinforcement Learning–A Tutorial. arXiv preprint arXiv:200604938. 2020.

22. Baldi, Simone and Rosa, Muhammad Ridho and Wang, Yuzhang. Model+ Learning-based Optimal Control: an Inverted Pendulum Study. 2020 IEEE 16th International Conference on Control & Automation (ICCA).

23. All the codes described in the manuscript are open-source and available at `github.com/francofusco/pendule_pi`. A reference manual is also published at `francofusco.github.io/pendule_pi` to assist teachers and students during the first-time hardware-setup.

24. Watkins CJCH. Learning from Delayed Rewards [Ph.D. thesis]. Cambridge University; 1989.

25. Watkins CJCH, Dayan P. Q-learning. Machine Learning. 1992;8:279–292.

26. See the Supplemental Material.

27. Haarnoja T, Zhou A, Hartikainen K, Tucker G, Ha S, Tan J, et al. Soft Actor-Critic Algorithms and Applications. arXiv:181205905. 2019.

28. Khalil W, Dombre E. Modeling, Identification and Control of Robots. Khalil W, Dombre E, editors. Oxford: Butterworth-Heinemann; 2002.

29. Mnih V, Kavukcuoglu K, Silver D, Rusu AA, Veness J, Bellemare MG, et al. Human-level control through deep reinforcement learning. Nature. 2015;518(7540):529–533.

30. Kingma DP, Ba J. Adam: A method for stochastic optimization. arXiv preprint arXiv:14126980. 2014.

31. Akiba T, Sano S, Yanase T, Ohta T, Koyama M. Optuna: A Next-generation Hyperparameter Optimization Framework. In: Proceedings of the 25rd ACM SIGKDD International Conference on Knowledge Discovery and Data Mining; 2019. .
